# Balancing Selection on *CDH2* May Be Related to the Behavioral Features of the Belgian Malinois

**DOI:** 10.1371/journal.pone.0110075

**Published:** 2014-10-10

**Authors:** Xue Cao, David M. Irwin, Yan-Hu Liu, Lu-Guang Cheng, Lu Wang, Guo-Dong Wang, Ya-Ping Zhang

**Affiliations:** 1 State Key Laboratory of Genetic Resources and Evolution and Yunnan Laboratory of Molecular Biology of Domestic Animals, Kunming Institute of Zoology, Chinese Academy of Sciences, Kunming, China; 2 University of Chinese Academy of Sciences, Beijing, China; 3 Kunming College of Life Science, University of Chinese Academy of Sciences, Kunming, China; 4 Department of Laboratory Medicine and Pathobiology, University of Toronto, Toronto, Ontario, Canada; 5 Laboratory for Conservation and Utilization of Bio-resource and Key Laboratory for Microbial Resources of the Ministry of Education, Yunnan University, Kunming, China; 6 Kunming Police Dog Base, Ministry of Public Security, Kunming, China; Kunming Institute of Zoology, Chinese Academy of Sciences, China

## Abstract

The Belgian Malinois (BM) is an excellent working dog that typically shows a circling behavior when placed in a confined space. Moreover, individuals showing moderate running in circles (one kind of obsessive compulsive behavior) in confined spaces typically show better work performance compared to those without the circling behavior or to those with a serious circling behavior (which can be defined as an obsessive compulsive disorder (OCD)). To determine whether the candidate gene *CDH2*, Cadherin 2, which is associated with OCD in the Doberman pinscher breed of dogs and in humans, was linked with this behavioral character in the BM, population genetic analyses were performed on a BM population and a natural population of the Chinese indigenous dog (CID). Many genetic signals of balancing selection were detected for one specific region of the *CDH2* gene, which suggests that a genomic block, which is included in the *CDH2* gene, experienced balancing selection in the BM, and that the *CDH2* gene might be associated with the behavioral characteristics of the BM dog (a balance between circling behavior and work performance). Moreover one specific variant, G63913941A, which creates a predicted transcription factor-binding site, may be the key mutation in the *CDH2* gene affecting the behavior of BMs by allowing the binding of a transcription factor and increasing *CDH2* expression.

## Introduction

The Belgian Malinois (BM), a military working dog with high excitement levels, commonly shows a circling behavior when in a confined space. In confined spaces that are not large enough to allow running, the BM will travel in circles (http://dogbreedsinfo.org/Belgian-Malinois.html). Moreover, in the BM population, individuals showing moderate running in circles (one kind of obsessive compulsive behavior) in confined spaces have better work performance, as they have a stronger desire and initiative for work than individuals that do not circle, and have better behavioral control (not showing extra circling behavior) than individuals that show extremely high levels of circling behavior.

This extreme circling behavior seen in the BM has been defined as an Obsessive-compulsive disorder (OCD) [1]. An OCD has been characterized in the Doberman pinscher breed of dogs, and was shown to have a highly significant genetic association with alleles of the Cadherin 2 (*CDH2*) gene region [2], [3]. Rare missense variants in *CDH2* are associated with OCD and Tourette disorder in humans [4]. These observations suggest that the *CDH2* gene may contribute to OCD; therefore, we speculated that variants in the *CDH2* gene might be involved in the circling behavior seen in the BM.

To investigate this question, population genetic analyses were performed on a BM population, which shows a balance in the behavioral characters of the circling behavior and work performance, and in a natural population of Chinese indigenous dogs, a local dog population that does not show this behavioral character. By comparing the population genetic signals from these two populations with differing behavioral characters, we should be able to determine whether the *CDH2* gene is associated with the balanced behavioral characteristics seen in the BM. Population genetic analysis identified signals of balancing selection in the *CHD2* gene in the BM population, which implies that the genomic block that includes the *CDH2* gene is experiencing balancing selection in the BM. This result suggests that the *CDH2* is involved in the behavior characteristics of circling and working in the BM. Moreover a variant (G63913941A) in a predicted transcription factor-binding site may be the key mutation in the *CDH2* gene that affects BM behavior by increasing the binding of two transcription factors to regulate expression of *CDH2*.

## Materials and Methods

### Ethics statement

Written informed consent for research purposes was obtained from the owners of the dog individuals used in this study. All samples used in this study were obtained and handled following the guidelines of the by-laws on experimentation on animals, and was approved by the Ethics and Experimental Animal Committee of Kunming Institute of Zoology, Chinese Academy of Science, China (KIZ_YP201002).

### Population Samples and Sequencing of *CDH2*

**Figure 1:**
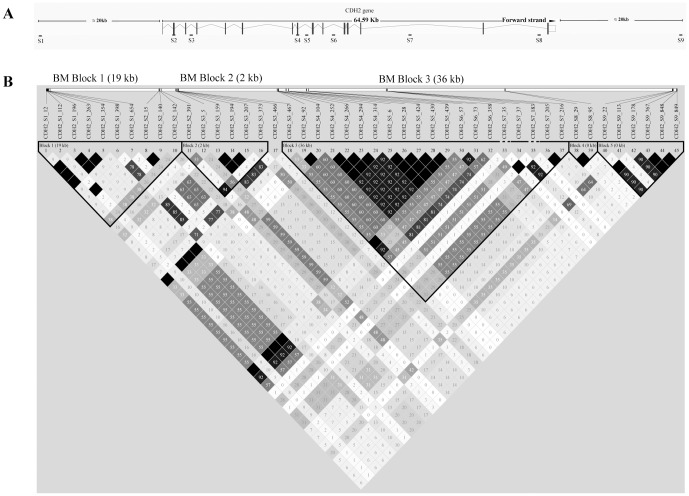
Locations of the sequenced segments and LD distribution in the *CDH2* gene. (A) Schematic map of the locations of the 9 sequenced segments in the *CDH2* gene region. (B) LD distribution in the *CDH2* gene in the BM population. Location to the LD Blocks in the BM (BM Blocks) is indicated in Fig. 1B.

Two dog populations, a Chinese indigenous dog (CID) and a Belgium Malinois (BM) population, were used for this study. The CID population consisted of 23 individuals while the BM population was composed of 53 individuals. DNA was extracted from blood samples according to a standard phenol-chloroform extraction procedure. Primers to amplify and sequence 7 segments within the *CDH2* gene, as well as one upstream and one downstream segment of the gene (Figure 1A) were designed based on the reference dog genome (canfam2). PCR products were sequenced on an ABI PRISM 3730xl analyzer (Applied Biosystems) with Big Dye Terminator sequencing Kits (v3.1 Applied Biosystems). Sequences were analyzed using DNASTAR software (DNASTAR). The sequences of segment S8 which is shorter than 200bp were provided in Sequence S1.

### Population genetic analysis

PHASE 2.1 [5], [6] was used to estimate the haplotype phase for every individual. We then used the Haploview program [7] to infer the Linkage disequilibrium (LD) parameters D′ and r^2^ and the LD blocks for each population. DNAsp 5.10.01 [8] was used to calculate the nucleotide diversity, θw and θπ, and to perform the Tajima's D test for each PCR segment. We used the “False Discovery Rate” (FDR) method of Benjamini and Hochberg [9] for multiple testing to correct the P values of Tajima's D. The PSMC approach was used to estimate the demographic history of dogs and wolves before 10,000 years ago with the parameters: -N25 -t15 -r5 -p “4+25*2+4+6” [10]. To improve the accuracy of inferred historical recombination events, sex chromosome sequences and loci with less than two thirds of the average depth were removed. Using the estimated and documented demographic history of the BM population, we used the program ms [11] to generate 10,000 independent replicated sample. For each segment used in the ms simulation, the same number of segregating sites (*seg_sites*) and same length for the segments (*l_seg*) as seen in the original data were used. POPGENE 3.11 [12] was used to calculate the heterozygosity of each SNP and a haplotype genealogy for the candidate block was inferred by constructing Median-joining networks with Network 4.5.1.6 [13] (http://www.fluxus-engineering.com/). The online transcription factor binding site prediction tool TFSEARCH [14] was used to predict DNA transcription factor binding sites in the reference and derived sequences of the candidate segments. Scores for evaluating the transcription factor binding predictions were calculated as follows: 100.0 * (‘weighted sum’ - min)/(max - min). Score above a threshold score of 85.0 (scores in the region (0∼100.0)) were considered to be true binding [14].

## Results

Using published Single Nucleotide Polymorphisms (SNPs) data [15], we identified 9 segments within or near the *CDH2* gene that had abundant variations for sequencing in this study. Of the 9 segments, 7 were within the *CDH2* gene (two exonic and five intronic sequences), as well as one upstream and one downstream segment of the gene (Figure 1A).The sequenced segments have a total length of about 4,213bp and are distributed across a ∼110kb region around the *CDH2* gene. A total of 53 BM and 23 CID individuals were sequenced for all 9 segments, which resulted in the identification of a total of 41 SNPs that were shared by both populations and an additional 2 SNPs that were specific to the CID population and 4 to the BM population.

**Figure 2:**
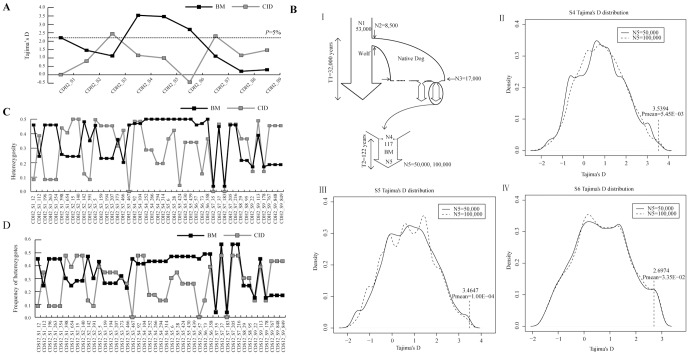
The distribution of three population genetic statistics and the information of coalescent simulations. (A) Distribution of Tajima's D values for the 9 segments of the *CDH2* gene region in the BM and CID populations. (B) Standard coalescent simulations under a neutral model for the genealogy of the BM. Part I is the genealogy of BM and parts II-IV are standard coalescent simulations for segments S4, S5, S6, respectively, under different values for N5 (50,000 and 100,000) in the BM population. P_mean_ is the mean of the P values for the Tajima D test. (C) SNP heterozygosity distribution in the *CDH2* gene region in the BM and CID populations. (D) Frequency distribution of heterozygotes for each SNP in the BM and CID populations.

In addition to calculating the population genetic parameters (Table 1), with a summary of the frequency spectrum at the polymorphic sites shown in Figure S1, we also inferred LD [7] and performed the Tajima's D test [8] for each segment of the *CDH2* gene region in both populations. Compared to the CID population, three much longer LD blocks (BM Block 1 about 19kb, BM Block 2 about 2kb and BM Block 3 about 36kb, Figure 1B) were found in the *CDH2* gene region in the BM population. In the CID population all LDs of the *CDH2* gene region were shorter than 500bp (Figure S2). A peak, which was within LD BM Block 3, having significant positive Tajima's D values (contained three segments S4: P = 5.01×10^−4^, S5: P = 6.95×10^−4^ and S6 P = 1.04×10^−2^), even after correction (S4: P =  4.51×10^−3^, S5: P =  3.13×10^−3^ and S6 P =  3.11×10^−2^), was observed in the Tajima's D distribution of the 9 segments in the BM population (Figure 2A). For the CID population, instead of a peak like that observed in the BM population, decreased values were observed in the Tajima's D distribution (Figure 2A). These results imply that the BM Block 3 region (contained the three segments S4, S5 and S6) likely was affected by balancing selection only in the BM population, however, it is possible that significantly positive values for Tajima's D may have been generated by demographic changes in the evolutionary history of the BM.

**Table 1:** Polymorphism statistics and neutral tests in both the BM and CID populations.

Population	Segments	Base pairs	N^1^	S^2^	Pi^3^	Theta-W
	S1	711	106	7	0.0037	0.0019
	S2	420	106	4	0.0032	0.0018
	S3	486	106	6	0.0042	0.0028
	S4	321	106	6	0.0092	0.0036
BM	S5	461	106	5	0.0055	0.0021
	S6	501	106	3	0.0029	0.0011
	S7	251	106	5	0.0058	0.0038
	S8	151	106	2	0.0029	0.0025
	S9	911	106	6	0.0014	0.0013
	S1	711	46	7	0.0022	0.0022
	S2	420	46	4	0.0029	0.0022
	S3	486	46	6	0.0055	0.0028
	S4	321	46	6	0.0062	0.0043
CID	S5	461	46	6	0.0041	0.0030
	S6	501	46	3	0.0011	0.0014
	S7	251	46	3	0.0057	0.0027
	S8	151	46	2	0.0049	0.0030
	S9	911	40	6	0.0024	0.0015

Note:

1 The number of chromosome; ^2^ Number of segregating sites; ^3^ Nucleotide diversity.

To rule out an influence of demographic history, we conducted simulations of the evolution of BM Block 3 (segments S4, S5 and S6), which shows significantly positive Tajima's D values, that following the demographic history of the BM dog [11] under a neutral evolutionary model. Like most other breeds of dogs, the demographic history of the BM dog can be divided into two stages: an ancient domestication period and a more modern breeding period, where the population experienced two bottlenecks, one during early domestication and a second during recent breed creation (Figure 2B (part I)) [16], [17]. We used three published individual genomes (BM: DogBM, CID: DogCI1, Wolf: GW3) to reconstruct the population history of dog [15] using the PSMC approach. The PSMC analysis showed that the ancient domestication period of the BM fit the published demographic history [15] very well (Figure S3). The dog was domesticated about 32,000 years ago, thus T1≈10,667 generations was used in our simulations, which assumes a generation time of 3 years. This research also indicated that the dog was domesticated from about 8,500 wolves whose effective population size was about 53,000, thus these numbers were also used in our simulations (N2 = 8,500 and N1 = 53,000). Wang and colleagues estimated that after domestication the effective population size for the domesticated dog increased to around 17,000 [15], and this number (N3 = 17,000) was used as the effective population size for the domesticated dog before the creation of the BM breed. The BM breed was created in 1891 from 117 domesticated dogs (http://www.fci.be/nomenclature/BELGIAN-SHEPHERD-DOG-15.html; http://www.akc.org/breeds/belgian_malinois/history.cfm) (thus T2≈41 generations and N4 = 117). BM dogs have now become important working dog and are used throughout the World with an estimated population effective size between 50,000 and 100,000. We therefore conducted simulations with differing values for N5 (50,000 and 100,000). A summary of the demographic history of the BM is shown in Figure 2B (part I). The ms simulation was performed with the following parameters: ms 106 1 -s *seg_sites* -G 31073.04049 -eG 0.0002 0.0 -eN 0.000205 0.34 -eN 0.05333 0.17 -eN 0.053335 1.06| perl ms_PopGen.pl 106 *l_seg*; ms 106 1 -s *seg_sites* -G 69077.55279 -eG 0.0001 0.0 -eN 0.0001025 0.17 -eN 0.026665 0.085 -eN 0.0266675 0.53| perl ms_PopGen.pl 106 *l_seg*.

Using the simulated demographic history and the parameters described above, the distributions of the Tajima's D obtained for the three sequence segments from the simulations are shown in Figure 2B (parts II-IV). From these simulations, more than 96% of the Tajima's D values are contained in the range (-∞ to 3), while less than 4% in the extreme positive range (3 to +∞). The probabilities, from these simulations, of obtaining the observed Tajima's D values for the experimental segments S4, S5 and S6, if they were neutrally evolving sequences, are 4.60×10^−3^, 1.00×10^−4^, 3.32×10^−2^ when N5 = 50,000, respectively, and 6.30×10^−3^, 1.00×10^−4^, 3.38×10^−2^ when N5 = 100,000, respectively (Figure 2B). The results from the simulations indicate that the significantly positive values for Tajima's D obtained from the BM sequences have an extremely low chance of being generated simply due to demographic history. These analyses suggest that balancing selection on BM Block 3 (covering a region of about 5501bp) of the *CDH2* gene likely explains the observed sequence pattern.

**Figure 3:**
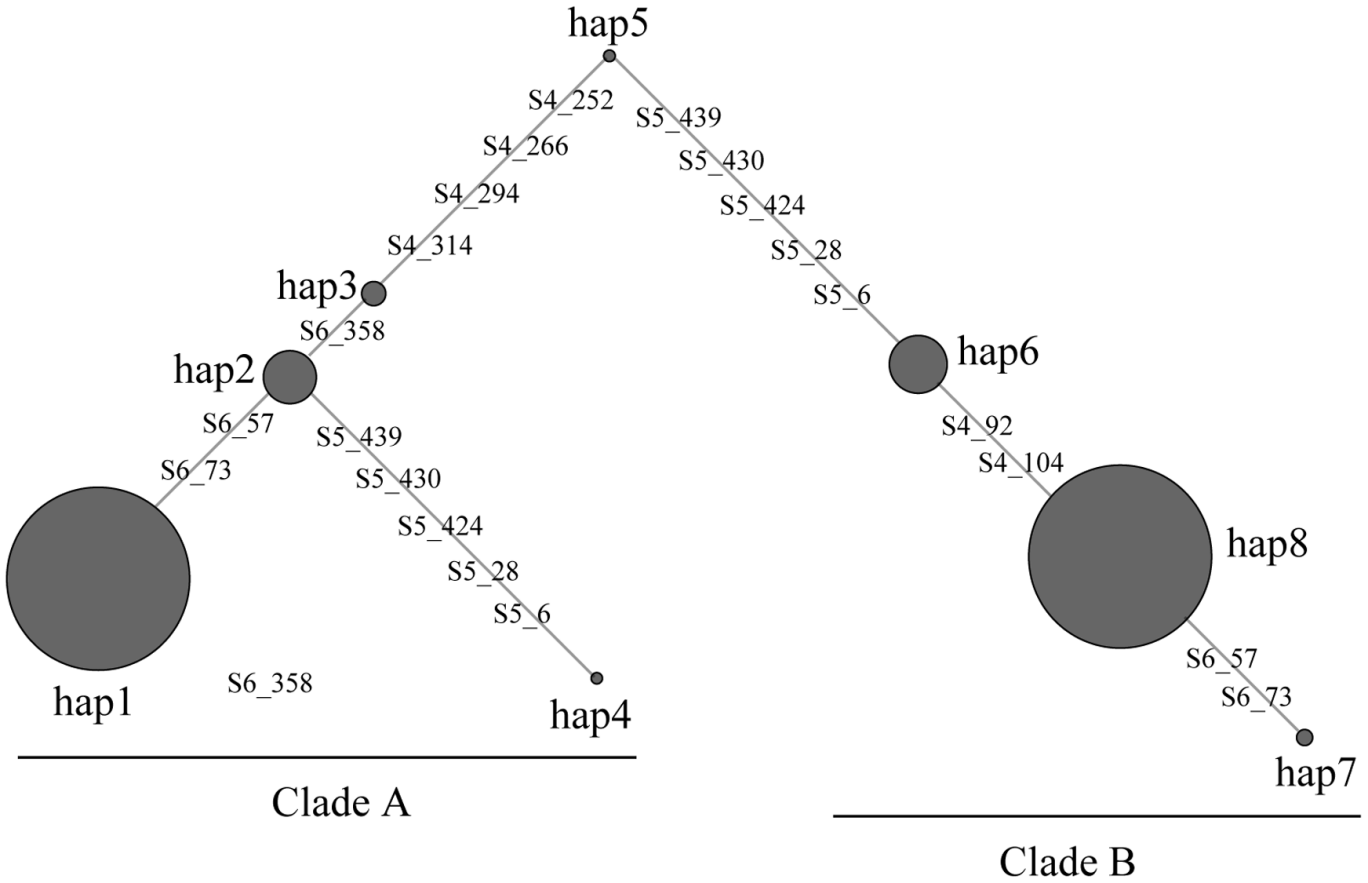
Median-joining network showing the genealogy of the BM Block 3 haplotypes in the BM population. Each haplotypes is shown as a circle with the size of the circle representing the haplotype frequency. Nucleotide differences between haplotypes are shown on the branches of the network.

Balancing selection maintains high frequencies of heterozygosity in sequences and results in haplotypes that show special genealogical patterns, therefore we calculated the heterozygosity [12] and the frequency of the heterozygotes for each SNP in the *CDH2* gene region in both populations, estimated the haplotype phase [5], [6] of the BM Block 3 loci in the BM population and generated a median-joining networks of the haplotypes [13]. A continuous distribution of SNPs with high heterozygosity values and frequency of heterozygotes were found for BM Block 3 (Figure 2C, Figure 2D) in the BM population. In contrast, in the CID population a wave distribution of SNP heterozygosity values and frequency of heterozygotes were observed (Figure 2C, Figure 2D). Moreover, in the median-joining network, the eight BM Block 3 haplotypes were distributed into two major clades (clade A: 50%, clade B: 49%) that were separated by a relatively long-branch length (Figure 3). These results support the conclusion that BM Block 3 of the *CDH2* gene experienced balancing selection.

To identify key SNPs within BM Block 3 that affected *CDH2* gene function we focused on 20 SNPs from published dog SNP data [15]. Of these 20 SNPs, 14 were only observed in the heterozygous state in the BM in the published data, and the derived state for these alleles were not observed in the three CID individuals where SNP information is contained in the published data [15]. The genomic locations of these 14 SNPs were obtained from the Ensembl genome database, which showed that all 14 are located within introns of the *CDH2* gene [18], [19]. As intronic regions typically do not directly affect protein products, we hypothesized that an intronic SNP may affect expression of the gene by changing the binding of a transcription factor. To examine this possibility, we used TFSEARCH [14] to predict the transcription factor DNA-binding sites in the reference and derived sequences of these three segments. Differences in four relevant mammalian transcription factor binding sites were found between the derived and reference sequences. The loss of binding sites for the sex-determining region Y gene product (SRY) were found in the derived sequences for SNPs (T63912191A, score: 90.0; T63916864C, score: 90.0 and T63916868C, score: 90.0), while gains of binding sites for SRY (score: 92.7), GATA-binding factor 2 (GATA-2) (score: 88.5), CCAAT/enhancer binding protein beta (*C/EBPβ*) (score: 88.0) and alpha (*C/EBPα*) (score: 85.7) were found in the derived sequences for the SNPs (A63912337G, G63912459Aand G63913941A). Since *CDH2* is mainly expressed in cardiac myocytes, smooth muscle and nervous system [20] and C/EBPβ and C/EBPα, which binds to the derived sequence containing SNP G63913941A, are also expressed in these same cells [21]–[23], we speculated that the variant G63913941A SNP may affect *CDH2* gene function. We then examined the allele frequency and the genotype distribution of this SNP in the BM and CID populations. A significantly (P<0.01, by χ^2^ test) higher derived allele (A) frequency exists in BM population (50%) compared to the CID population (27.78%). Moreover, the genotype distribution in the BM population were also significantly different from that in the CID population (P<0.01, by χ^2^ test). In the BM population, the AG genotype was the most frequent genotype (GG 26.42%, AA 26.42%, AG 47.16%), while GG was most frequent in the CID population (GG 65.72%, AA 8.70%, AG 26.09%).

## Discussion

Here we used population genetics methods to analyze variants in the *CDH2* gene in populations of the BM (where most individuals show the circling phenotype) and in the CID (a natural population of dogs). Differences in the LD distribution pattern of the *CDH2* gene between the BM and CID populations, and results from the Tajima's D test indicate that BM Block 3 of the *CDH2* gene may have experienced balancing selection in the BM population or have been affected by the demographic history of the BM. Simulations of the demographic history of the BM eliminate the possibility that the LD distribution pattern or Tajima's D test results are due to the demographic history of the BM. Moreover, the levels of heterozygosity and frequency of heterozygotes across BM Block 3 and the structure of the median-joining networks of the haplotypes offer additional support for a hypothesis of balancing selection.

Balancing selection has been shown by previous research [24]–[26], to maintain polymorphisms and produce individuals that have a balance of advantageous and disadvantageous characteristics. For instance, balancing selection has been shown to occur on the myostatin (*MSTN*) gene in the whippet breed of dogs, where individuals carrying only one copy of the mutated allele of *MSTN* (with a medium muscular phenotype) are significantly faster in competitive racing than individuals carrying the wild-type genotype (showing less muscularity) and are of better physical appearance than individuals carrying two copies of the mutation (which a greater level of muscularity) [25]. Similarly, in the BM population, BM dogs that run in circles in confined spaces at an intermediate level have the better work performance than those that do not circle or those that circle at a high level (which is considered to be OCD) [1]. Our results showing that BM Block 3 of *CDH2* (an OCD relevant gene) experienced balancing selection in the BM population suggests that the BM Block 3 of the *CDH2* gene is associated with the behavioral characteristics of the BM dog (a balance between circling behavior and work performance).

The derived sequence suggests a possible mechanism for the circling phenotype. A change in the potential to bind the transcription factor (*C/EBPβ*) was found for the derived *CDH2* sequence, similar to an inferred change in predicted transcription factor DNA-binding in Alzheimer's disease [27], which is also associated with OCD [28], [29]. The frequency of the derived allele (containing an additional *C/EBPβ* binding site) was significantly higher in the BM population, which shows circling behavior, than in CID population, which does not show the circling behavior. These results suggest that the heterozygous genotype was advantageous in the BM population, allowing some circling in confined spaces, but not to a level that seriously affects work performance. The variant G63913941A may be the key mutation in the *CDH2* gene for BM behavior, which generates a *C/EBPβ*binding site that may have allowed increased expression of this gene and ultimately affects both circling and work behavior.

Our work revealed a strong signal for balancing selection in a genomic block of the *CDH2* gene in the BM population that shows a balance between a circling behavior and work performance. Since extreme circling is defined as an OCD [1] our results not only suggest that *CDH2* is associated with the behavioral characteristics seen in the BM, but also imply that *CDH2* may related to other kinds of obsessive compulsive behaviors found in dog populations. A noncoding variant in *CDH2* may be the key mutation correlated with the behavioral character (balancing of circling behavior and work performance) of the BM. These results are similar to those of Tang et al. [3] who found functional noncoding variants that affect transcription factor binding in a different canine model of OCD. These results hint that noncoding sequences play important roles in OCD, thus greater attention to regulatory sequence may be necessary in OCD research.

## Supporting Information

Figure S1 **Frequency spectrums of polymorphic sites in two populations.** (A) Frequency spectrums of polymorphic sites in BM population. (B) Frequency spectrums of polymorphic sites in CID population.
(PDF)

Figure S2 **LD distribution in the** ***CDH2*** **gene of the CID population.**
(PDF)

Figure S3 **Demographic history of dogs and wolves before 10,000 years ago estimated by PSMC.**
(PDF)

Sequence S1 **The sequences of segment S8 which is shorter than 200bp.**
(RAR)
