# Supplementary figures and images for: Balancing Selection on CDH2 May Be Related to the Behavioral Features of the Belgian Malinois
Source: PLoS One. 2014 Oct 10;9(10):e110075. doi: 10.1371/journal.pone.0110075 (PMC4193869; doi:10.1371/journal.pone.0110075)

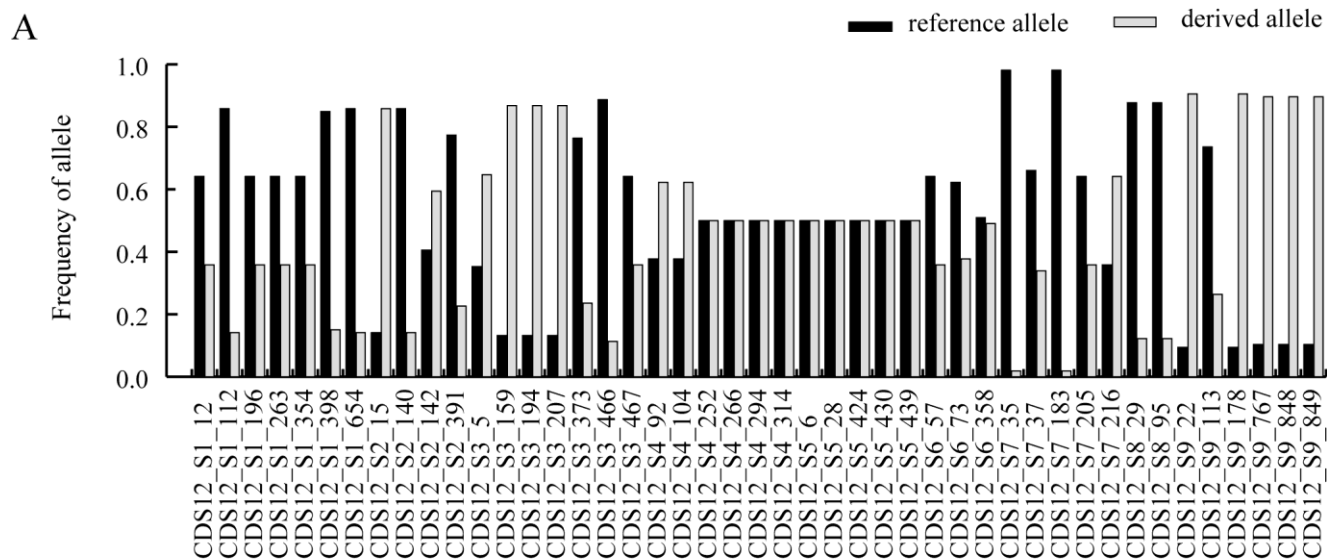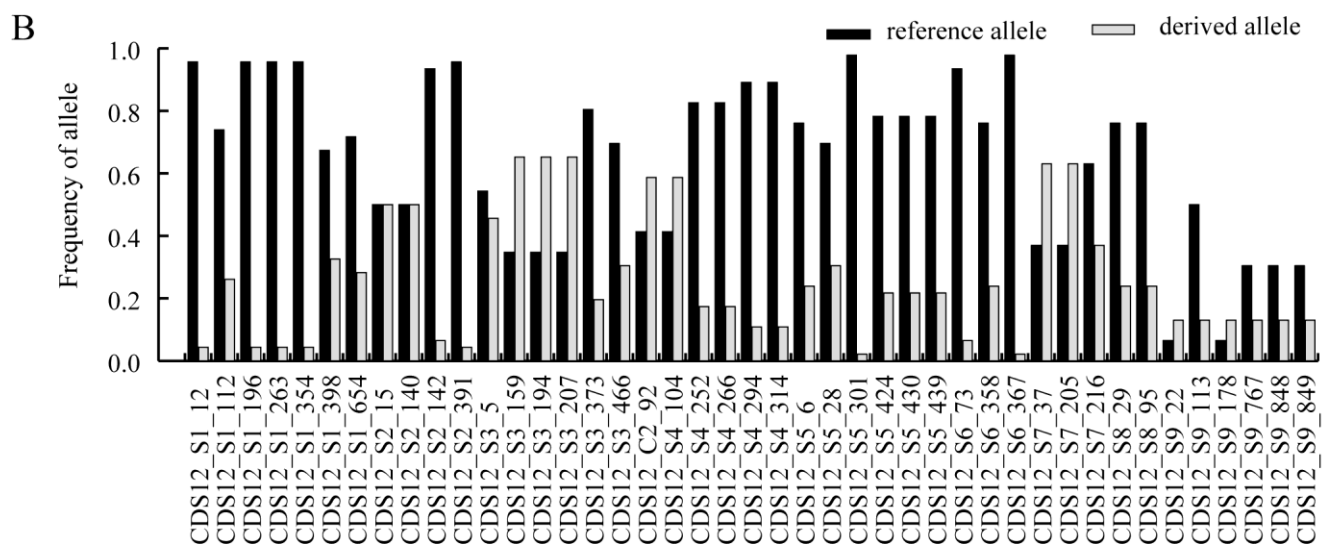

Supplement: Figure S1 — Frequency spectrums of polymorphic sites in two populations. (A) Frequency spectrums of polymorphic sites in BM population. (B) Frequency spectrums of polymorphic sites in CID population. (PDF) [file pone.0110075.s001.pdf]

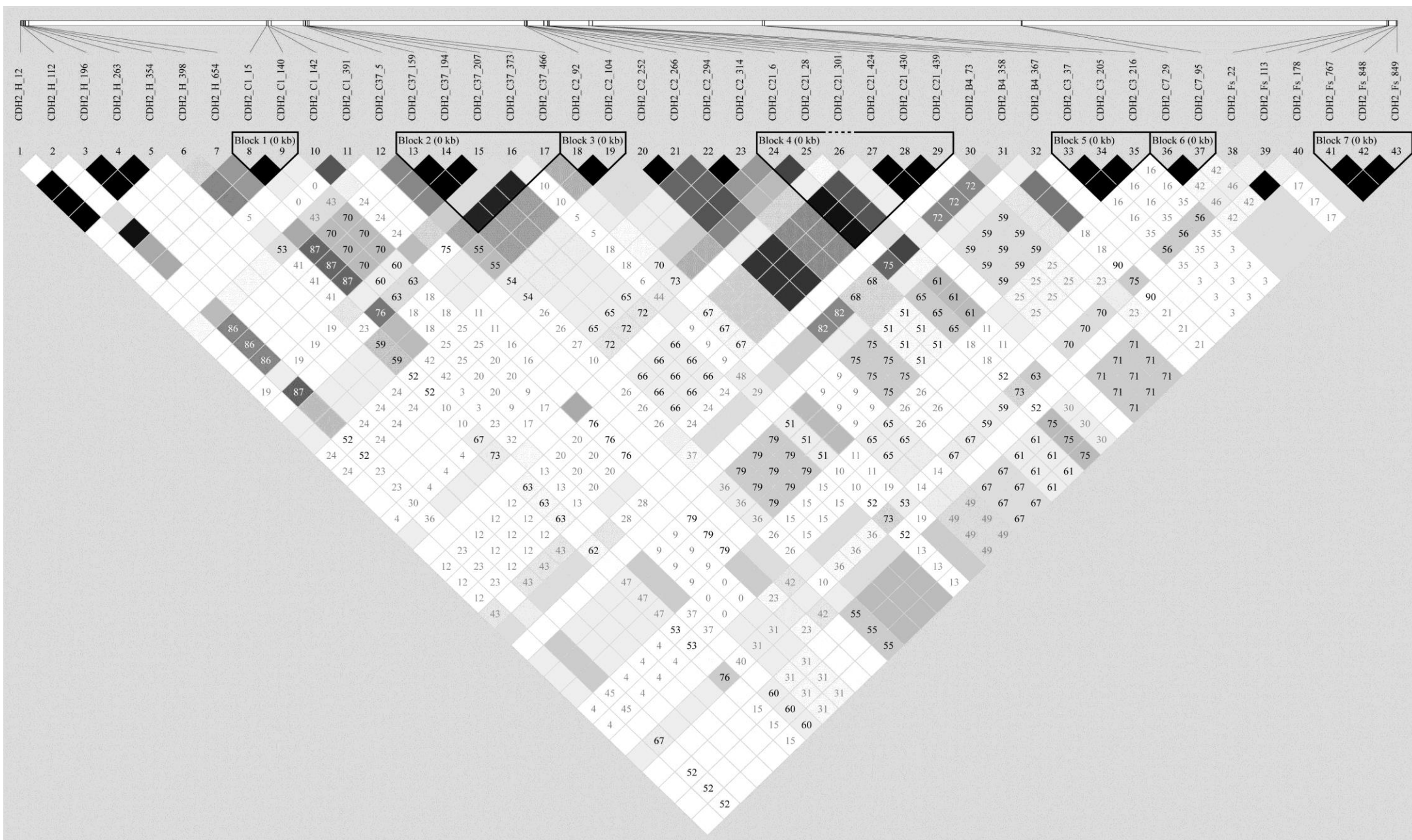

Supplement: Figure S2 — LD distribution in the CDH2 gene of the CID population. (PDF) [file pone.0110075.s002.pdf]

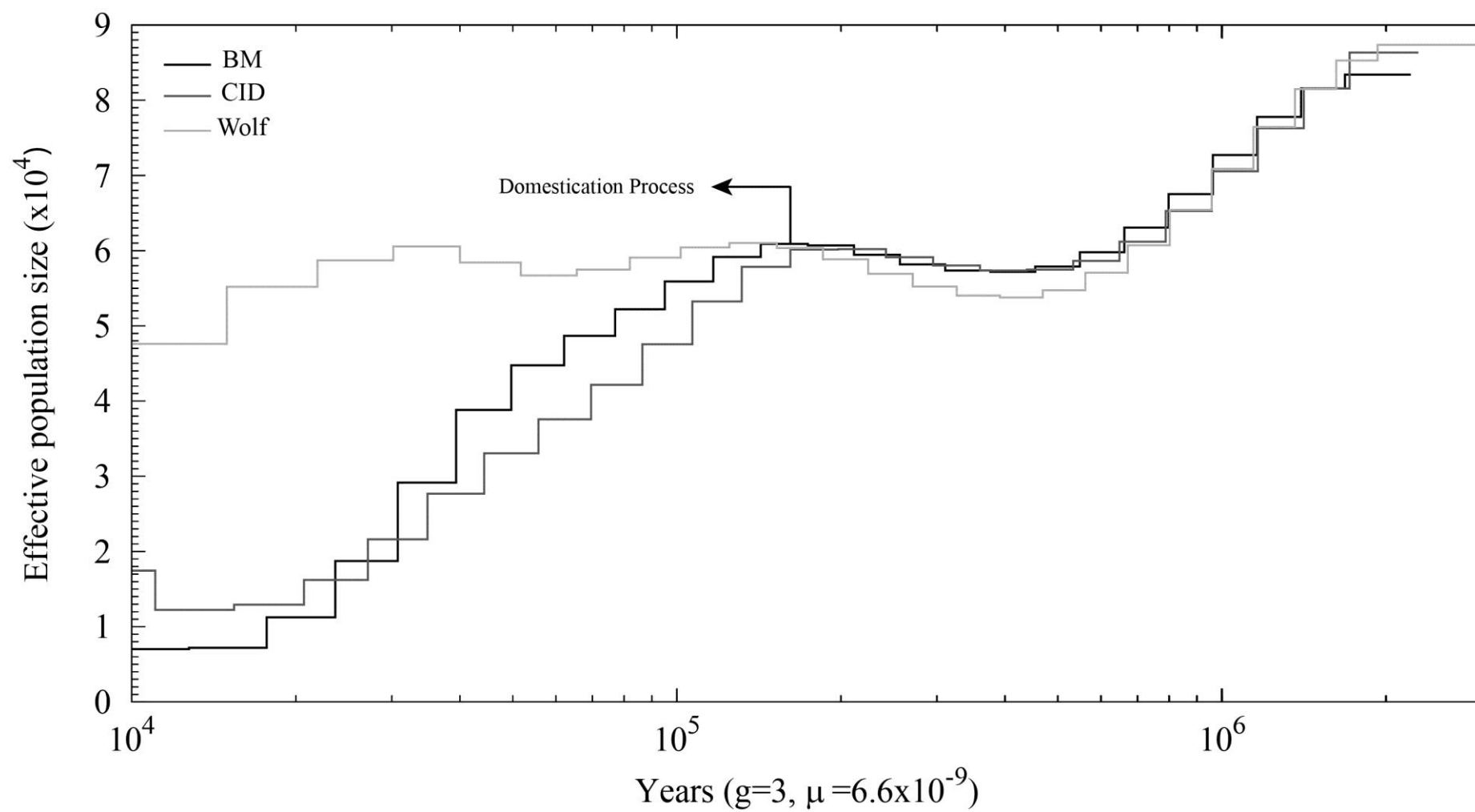

Supplement: Figure S3 — Demographic history of dogs and wolves before 10,000 years ago estimated by PSMC. (PDF) [file pone.0110075.s003.pdf]
